# Self-reported unemployment status and recession: An analysis on the Italian population with and without mental health problems

**DOI:** 10.1371/journal.pone.0174135

**Published:** 2017-04-04

**Authors:** Fabrizio Starace, Francesco Mungai, Elena Sarti, Tindara Addabbo

**Affiliations:** 1 Department of Mental Health & Drug Abuse, AUSL Modena, Modena, Italy; 2 Department of Economics University of Modena and Reggio Emilia, Modena, Italy, Marco Biagi Foundation; University of Rijeka, CROATIA

## Abstract

**Purpose:**

During economic recession people with mental health problems have higher risk of losing their job. This paper analyses the issue by considering the Italian rates of unemployment amongst individuals with and without mental health problems in 2005 and 2013, that is prior and during the economic crisis.

**Methods:**

We used data from the National surveys on “Health conditions and use of health services” carried out by the Italian National Institute of Statistics (ISTAT) for the years 2005 and 2013. The surveys collected information on the health status and socioeconomic conditions of the Italian population. Self-reported unemployment status was analysed amongst individuals with and without reported mental health problems. In addition, descriptive statistics were performed in order to detect possible differences in the risk of unemployment within different regional contexts characterised by different socio-economic conditions.

**Results:**

The recession determined increased disparities in unemployment rates between people with and without mental health problems. Regardless to the presence of mental health problems, young people were more likely to be unemployed. Among people who reported mental health problems, males were more likely to be unemployed than females. People with low education level were more likely to be unemployed, particularly during the recession and in presence of mental health problems. Changes in unemployment rates due to the crisis showed different patterns across different regions of the Country.

**Conclusions:**

These analyses confirm that in periods of economic crisis people with mental health problems are at risk of experiencing exclusion from labour market. In addition, the impact is even worse within the group with low education and younger age. These findings emphasise the importance of specific interventions aimed at promoting labour market participation and reintegration for people with mental health problems.

## Introduction

Unemployment represents a condition significantly associated with poorer physical and psychological health [1]; in addition, it has been documented that being unemployed during periods of financial crisis is particularly detrimental both to physical and mental health [2]. Over the last decade, as a result of the recent Great Recession, uncertainty of labour market and progressive reduction of job opportunities have been paramount problems. Distress deriving from personal debt, isolation, social exclusion secondary to job loss, are known to be significant determinants of mental health conditions [1]. Unemployment has been also recognised to weaken the effectiveness of interventions targeted at mental health problems (MHP), thus favouring the persistence of illness and placing an even heavier burden on society [3, 4] and represents a significant hurdle against attainment of full personal recovery in people with mental health problems [5, 6].

On the other hand, suffering from mental illness during a period of financial crisis has been reported to represent a major risk of losing jobs; and, as the labour market becomes tight, it may be more difficult for subjects with MHP to access employment [7]. Moreover, economic recession tends to widen the social divide between people affected by mental illness and general population [8–12]. As a matter of fact, large-scale population studies reported that “unemployment” was strongly associated with a deterioration of mental health scores [13, 14].

Finally, the stigma associated to mental illness, has been postulated to represent an additional detrimental factor, which might get harsher during periods of economic downturn [9].

On the other hand, possible protective, mitigating factors on mental health, during spells of macroeconomic crisis, have been identified. State support and public services, for example, may exert a “shock-absorber” effect [15]. Accordingly, Countries with higher and favourable scores in mental health are those ensuring the strongest social welfare networks [16]. These findings emphasise the importance of social policies and the risk of cutting back on mental health care and social welfare measures, especially during times of economic hardship [16, 17].

In this paper we replicate, within an Italian sample, the analysis of a previous study, which assessed unemployment rates among individuals who reported MHP across 27 European Countries using data, retrieved from Eurobarometer 2006 and 2010 [9]. Authors reported that spells of financial hardship increase the likelihood of social exclusion in people reporting MHP and this was particularly true for individuals with lower education and males.

We investigated the impact of the economic crisis on the self-reported unemployment status in subjects with and without MHP in Italy. Among those subject reporting MHP we aimed to identify subgroups of people that might be particularly liable to the effects of the economic downturn.

We also assessed whether the impact of economic recession and unemployment rates produced different results in people who reported MHP living in different regions of Italy. Indeed, it has been argued that the impact of financial crisis is not always uniform across different Countries and it may depend on several variables such as, duration and severity of the crisis, austerity measures introduced by Governments and the level of social protection schemes available [18]. Several recent studies report that, in terms of welfare systems, significant heterogeneity exists even within the same Country, as regional authorities have gained major responsibilities on control and implementation of social policies [19]. According to Bertin and Carradore Italian welfare system is no exception, it is far from being homogeneous and the resources allocated at regional level have deemed to affect considerably the social services provided [20]. Thus, we explored whether these differences may have specifically affected regional labour markets in Italy and, if so, whether this data may reflect inequalities in unemployment rates between people with and without MHP.

## Materials and methods

### Data source

Data were retrieved from the Italian National Institute of Statistics (ISTAT) surveys ‘Health Conditions and Use of Health Services’, carried out in 2004–2005 and 2012–2013 in different samples, albeit highly statistically representative, of the Italian population [21]. The surveys provided information on the health status and socio-economic conditions of the Italian population and reported socio-demographic data, health status and working conditions at regional level.

The 2004–2005 survey included 50,474 households and 128,040 individuals; the 2012–2013 survey counted a total of 49,811 households and 119,073 individuals.

For the purpose of this analysis, we restricted the sample to adults of working age (i.e. 18–64 years), thus the 2005 and 2013 samples included 80,661 and 72,476 subjects, respectively. Sampling procedure ensured that results are representative of the whole Italian population.

### Assessments

General health and mental health indicators were measured by means of the SF-12 (12-Items Health Status Survey-Short Form 12) [22–26]. The SF-12 enables to investigate two concise indexes: PCS Physical Component Summary for physical health and MCS Mental Component Summary for mental health. Both indexes range from 0 to 100; higher scores at PCS and/or MCS are associated with better physical and mental health (e.g. no disabilities, elevated vitality, positive psychological attitudes, absence of psychological distress, no limitation in functioning due to mental illness). According to Kiely and Butterworth [26] a cut-point score of ≥40 on MCS-12 was adopted to discriminate between subjects with and without MHP. Unemployment status was defined as “looking for first or new job”.

To evaluate the consistency between the self-reported unemployment rates and actual unemployment rate official data reported by ISTAT in 2005 and 2013 were considered. According to ISTAT the Italian unemployment rates for the age group 15–64 was 7.8% in 2005 and 12.3% in 2013 [27]; in our sample, the self-reported unemployment status in the same age group was 7.2% in 2005 and 13.2% in 2013.

However, due to the definition adopted by ISTAT, the dataset does not include “inactive” subjects who might have given up looking for an occupation as consequence of lower chance of finding a job.

### Statistical analysis

Descriptive statistics for subjects with and without MHP were performed in both 2005 and 2013 samples. Moreover, we calculated the variation in rates of unemployment, prior and during the crisis, in people with and without MHP, for each Region of Italy to explore whether local welfare policies and socio-economic conditions might associate with different unemployment figures between the two groups.

Four separate multivariate logistic regression models were carried out to evaluate the ORs of the association between socio-demographic variables and unemployment in subjects with and without MHP both in 2005 and 2013. The explanatory variables in each model were: age groups (using the following age groups: 18–29, 30–39, 40–49 and 50–64, the latter being the reference group), gender (dummy variable taking the value of 1 if female), the presence of children (dummy variable taking the value of 1 if with children), civil status (dummy variable taking the value of 1 if married), education level (categorical variable with five categories where having a degree or more was the reference group), Region of residence (North-West, North-East, Centre and South/islands, the second being the reference group). Finally, a backward stepwise analysis was performed to assess the variables taken into account in the explicative model for predicting unemployment. The algorithm chooses to drop the least significant variable and then reconsiders all dropped variables (except the most recently dropped) for being reintroduced into the model. Two separate significant levels were chosen: p< .10 for deletion from the model and p< .01 for adding to the model.

All analyses were carried out using Stata version 13.

### Ethics statement

Ethical approval was not required as this study was performed as secondary data analysis on anonymous datasets.

## Results

Descriptive statistics and socio-demographic characteristics of ISTAT 2005 and 2013 samples are summarized in Table 1.

**Table 1:** Descriptive statistics among people with and without mental health problems aged 18–64 years old in ISTAT 2005 and 2013.

	2004–2005			2012–2013		
Variable	MH + (N 10200)	MH- (N 70461)	p	MH + (N 10728)	MH- (N 61748)	p
Unemployed	9.0%	7.1%	<0.001	18.2%	12.9%	<0.001
Female	62.9%	48.9%	<0.001	59.0%	49.4%	<0.001
**Age groups**						
18–29	17.4%	22.5%	<0.001	15.4%	20.1%	<0.001
30–39	20.2%	24.7%	<0.001	16.6%	21.0%	<0.001
40–49	23.7%	23.8%	0.710	26.2%	26.3%	0.878
50–64	38.8%	28.9%	<0.001	41.7%	32.7%	<0.001
With Children	73.5%	76.1%	<0.001	71.3%	72.6%	0.004
Married	58.1%	58.4%	0.674	53.4%	53.9%	0.348
**Educ. Level**						
Univ. degree or +	8.8%	11.3%	<0.001	11.4%	14.2%	<0.001
High school	34.4%	39.3%	<0.001	41.4%	45.5%	<0.001
Sec. school	33.4%	34.5%	0.038	34.9%	32.9%	<0.001
Primary school	19.3%	12.6%	<0.001	10.5%	6.7%	<0.001
No education	4.1%	2.4%	<0.001	1.9%	0.8%	<0.001
**Area**						
North-West	20.8%	21.3%	0.300	20.5%	22.0%	0.001
North-East	20.1%	20.1%	0.974	19.9%	19.9%	0.884
Centre	19.2%	17.3%	<0.001	18.1%	17.4%	0.080
South/Islands	39.9%	41.4%	0.006	41.5%	40.6%	0.096

The sample of subjects between 18–64 years showed a balanced gender distribution in the group without MHP, whilst females were more represented (62.9% in 2005 and 59% in 2013) among those people who reported MHP. The mean age was 41 for the subjects without MHP (more specifically, it was 42 in 2005 and 40 in 2013) and it was 44 in 2005 and 43 in 2013 for individuals who reported MHP. People between 18 and 39 years of age were significantly more represented in the group without MHP (p = 0.000 both in 2005 and 2013), whilst people of older age were more frequent in the subsample of subjects reporting MHP.

The rate of unemployed subjects was significantly higher in subjects who reported MHP in both surveys, yet reaching the highest level in 2013 (18.2%).

The majority of people interviewed was married and had children, even though the total of married people was less in 2013 in both samples (with and without MHP). However, the marital status was not significantly different between the two groups in both years. People without MHP had, on average, higher education levels. As regards to education level, significant differences were found by mental health status in both 2005 and 2013 sample. People with no education or a primary school level were more represented in subjects who reported MHP, especially in 2005. Highest levels of education were more frequent in people without MHP, mainly in 2013. Finally, we found that the distribution of the population by mental health status within the regional areas presented with statistically significant differences in the Centre (p = 0.001) and South/Islands (p = 0.006) in 2005 and in the North West (p = 0.001) in 2013.

**Fig 1:**
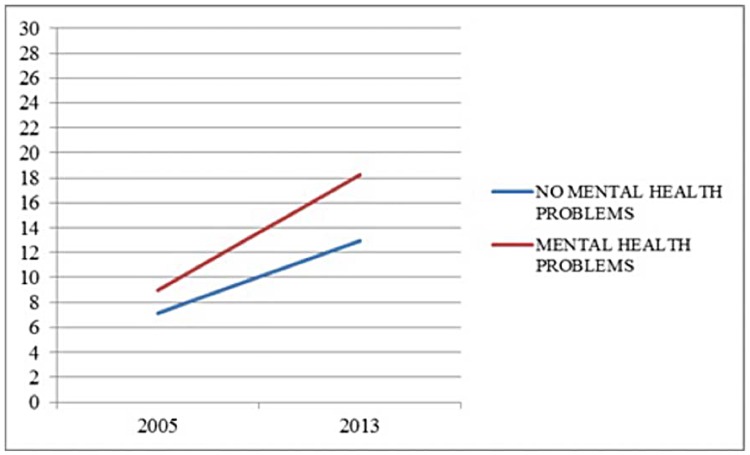
Self-reported unemployment status among individuals with and without mental health problems (aged 18–64 years old) in ISTAT 2005 and 2013.

As shown in Fig 1, the status of “being unemployed” was more frequent amongst people who reported MHP (9% in 2005 and 18.2% in 2013) compared with those without MHP (7% in 2005 and 13% in 2013) in both surveys. Overall, the percentage of people reporting to be unemployed was higher in 2013 than in 2005. The gap between those without MHP in the two years considered was 5.6%, meaning that during the crisis a considerable number of people had lost their job. However, the gap resulted to be wider for those people who reported MHP, reaching 9.3%.

The analysis, performed separately for the 20 Italian Regions, showed consistently that the overall rate of self-reported unemployment for people reporting MHP was higher compared with people without MHP, both in 2005 and 2013. A worsening in self-reported unemployment from 2005 to 2013 was found in the two groups, but in most of the Regions the strongest impact was on the group who reported MHP. The regional level analysis, allowed to identify four distinctive patterns, characterising different regional areas, which are likely to be related to different socio-economic conditions and local welfare policies. Self reported unemployment status among individual with and without mental health problems in 2005 and 2013 for each individual Region is showed in Appendix (S1 Appendix).

Results from the multivariate regression for predictors of unemployment by mental health status in 2005 and 2013 are reported in Table 2.

**Table 2:** Multivariate logistic regression analyses for predictors of unemployment by presence of mental health problems in ISTAT surveys 2005 and 2013.

	2004–2005				2012–2013			
	No mental health problems		Mental Health problems		No mental health problems		Mental Health problems	
Unemployment	Odds Ratio	[95% Conf. Int.]	Odds Ratio	[95% Conf. Int.]	Odds Ratio	[95% Conf. Int.]	Odds Ratio	[95% Conf. Int.]
Female	1.118***	[1.043; 1.199]	.625***	[.531; .735]	1.108***	[1.048; 1.170]	.703***	[.627; .788]
**Age Groups**								
50–64	1.000		1.000		1.000		1.000	
18–29	5.144***	[4.451; 5.944]	3.088***	[2.277; 4.187]	3.160***	[2.862; 3.489]	2.376***	[1.931; 2.924]
30–39	3.518***	[3.072; 4.029]	3.297***	[2.539; 4.282]	2.405***	[2.196; 2.633]	2.361***	[1.982; 2.813]
40–49	1.924***	[1.662; 2.229]	2.455***	[1.907; 3.161]	1.566***	[1.429; 1.715]	1.805***	[1.549, 2.105]
With Children	1.510***	[1.362; 1.673]	.975	[.795; 1.196]	1.325***	[1.233; 1.423]	1.095	[.952; 1.260]
Married	.388***	[.354; .426]	.474***	[.390; .575]	.504***	[.470; .541]	.611***	[.532; .701]
**Education**								
Degree or Higher	1.000		1.000		1.000		1.000	
High Sc.	.842**	[.748; .948]	.978	[.714; 1.34]	1.258***	[1.149; 1.378]	1.246**	[1.007; 1.541]
Sec. Sc.	1.200**	[1.065; 1.350]	1.637**	[1.208; 2.218]	1.912***	[1.743; 2.097]	2.099***	[1.698; 2.595]
Prim. Sc.	1.502***	[1.277; 1.768]	1.360*	[.948; 1.951]	1.861***	[1.607; 2.155]	2.157***	[1.650; 2.821]
No educ	1.454**	[1.126; 1.878]	.963	[.571; 1.622]	2.344***	[1.702; 3.228]	1.564*	[.972; 2.516]
**Area**								
North-East	1.000		1.000		1.000		1.000	
North West	.975	[.839; 1.133]	1.528*	[1.124; 2.077]	1.110**	[1.003; 1.223]	1.071	[.879; 1.305]
Centre	1.626***	[1.411; 1.873]	2.000***	[1.466; 2.727]	1.358***	[1.226; 1.507]	1.431***	[1.174; 1.744]
South/Isl.	3.801***	[3.399; 4.250]	4.041***	[3.119; 5.237]	2.476***	[2.282; 2.687]	1.916***	[1.625; 2.259]
**Const**.	.0123***	[.010; .015]	.029***	[.019; .045]	.035***	[.031; .041]	.090***	[.069; .117]
**Obs.**	70461		10200		61748		10728	

* p < 0.10,

** p < 0.05,

*** p < 0.01

Among people reporting MHP, males were more likely to be unemployed than females in both years, whilst the opposite was observed in the group of people without MHP.

Both in 2005 and in 2013, individuals falling within the youngest age groups (18–29 and 30–39), with and without MHP, were more likely to be unemployed than individuals in the oldest age group (50–64 years; reference group). However, age-related unemployment in both surveys was different among those with and without MHP; e.g. a younger age was more likely to be associated with higher unemployment rate amongst those without MHP (p<0.01).

People with low education levels were more likely to be unemployed and this was particularly true in 2013 for people without MHP (p<0.01); (reference group is, in this case, degree or a higher education level).

Living in the South/Islands areas of Italy was associated with a higher risk of being unemployed in both years, although a smaller gap was observed in 2013 with respect to other areas (p<0.01) (Reference group is the North-East).

Finally, a backward stepwise regression analysis was carried out, including in the model, as predictive factors, all the variables considered in the descriptive statistics (S2 Appendix).

The analysis performed on 2005 subjects without MHP showed that all variables had a p < 0.1000 (no variable removed); the most significant on unemployment were education level and area of residence, while the least influential was the civil status. As far as subjects who reported MHP were concerned in 2005, the variable about having children was removed from the model, as its p-value was above the significant level related to the probability for removal (i.e. p = 0.9067). The most significant variables resulted to be place of residence and education level, while the least were civil status and gender.

The analysis performed on the 2013 sample, again, kept all its variables in the model (i.e. all of them had a p-value < 0.1000) when considering the population without MHP, with the most significant being place of residence, presence of children and education level, and the least influential being civil status. When applied in the group who reported MHP, the variable having children was removed from the model (p-value equal to 0.1334). The ones with the highest impact on unemployment were education level and area of residence, while civil status and gender had the lowest impact.

## Discussion

To date, the causal link between unemployment and physical/psychological well-being in the general population remains a complex and controversial matter of debate. If on one hand, it is true that health indicators (both physical and mental) are negatively affected by long term insecurity due to unemployment, debt, social exclusion, inequalities of living standards (i.e. housing, nutrition) [1, 2], on the other hand, it has also been reported that other well-being indicators, such as “life satisfaction”, may not be affected during periods of high unemployment [28]. In fact, according to this hypothesis, the reduction of time at work and the availability of time for other activities (i.e. exercise and healthy dieting) may reduce the level of stress-related health deterioration and favour the adoption of a healthier lifestyle [28].

However, from a psychosocial point of view, evidence available supports the notion of unemployment being a determinant of psychological distress/MHP. The postulate in this case is that stress, due the consequences of unemployment (i.e. poverty, debt, deterioration of life standards and social status), has a detrimental impact on psychological well-being and consequently the risk of MHP [2, 29–31].

Moreover, a major challenge is represented by the difficulty of disentangling the association between the variables “unemployment” and “MHP” and operationalise them as either dependent or independent variables. Notwithstanding this dilemma, it remains undeniable that the combination of being unemployed with MHP has significant detrimental effects on people.

To our knowledge this is the first study carried out in Italy to investigate the association of economic crisis and MHP, as measured by the self-reported unemployment status.

The study confirms the findings reported by Evans-Lacko et al. [9] regarding the association between periods of economic downturn and the increased likelihood of unemployment in people who reported MHP; this is particularly true if we look at most vulnerable segments of our society (e.g. people with lower education level, male subjects).

So far, many studies have documented that economic recession had a negative impact on employment conditions, but there are still limited data available regarding specific vulnerable sub-groups, on which adverse external conditions can cause worse effects.

Overall, the present analysis gives further evidence, within an Italian nationally representative sample, of the negative impact of economic crisis on society as a whole, in terms of psychological distress. The consequences of the economic downturn have been worse within certain subgroups of the general population. More specifically, our data on unemployment showed more adverse consequences in people who reported MHP, with lower education levels and living in the South/Islands Regions.

Therefore, our data support the notion that economic crises tend to strike with disproportionate violence individuals belonging to the most vulnerable segments of our society.

Several possible explanations for this phenomenon can be considered. During economic downturns the job market becomes tight with a high firing rate and lower re-employment chance for those with greater disadvantages. There also may be shifts in the labour market demand, potentially affecting specific subgroup of the population; in this regard, Seguino reported that at the beginning of the crisis, when the industries more exposed to the downturn are commonly construction and manufacturing, a higher presence of men employed in these sectors may be affected, leading to an initial harsher effect in men [32].

An additional problem is represented by the fact that individuals with MHP are often labelled and discriminated against. Available data suggest that stigma towards people with mental illness is a potentially major determinant of unemployment, particularly during times of economic recession. The persistence of stereotypes related to unproductivity and unpredictable behaviour linked to mental illness and the subsequent marginalisation in society represents a major problem [33]. As reported by Warner [34] and more recently by Evans-Lacko [9], attitudes towards mental illness may become even harder during spells of crisis.

We believe that our findings should prompt a serious analysis and planning of specific interventions aimed at promoting labour market participation for people with MHP, especially during times of economic recession. Likewise, evidence on the impact of recession on vulnerable subgroups, such as individuals with lower education level and suffering from MHP should be the focus of dedicated research and dealt with via specific policies and welfare measures.

Available evidence seems unequivocal regarding the opportunity to maintain and/or provide sufficient resources to Health and Social Services, in order to keep or reintegrate people in employment, as this appear to be the most effective measure to counterbalance the negative effect of the crisis on health [35].

Programmes promoting mental health are even more important during times of recession. Regrettably, progressive cuts in mental health funding all across Europe [9] and Italy as well, has been reported so far. This is straining mental health and social services, leaving health providers under the pressure of a greater demand with reduced resources. In this respect, our findings showed that Italy is not dissimilar to other Countries of the Eurozone when facing the consequences of the economic downturn.

**Fig 2:**
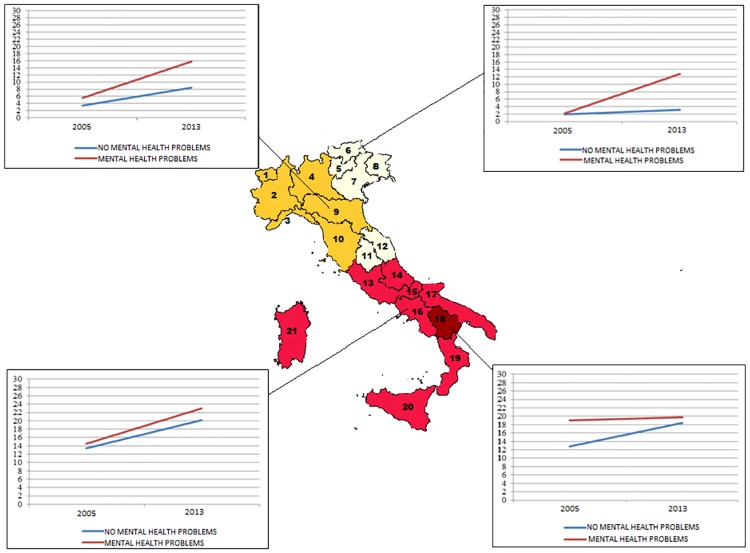
Four different patterns were identified across Italy according to self-reported unemployment status among individuals with and without mental health problems (aged 18–64 years old) in ISTAT 2005 and 2013.

In addition, the analyses carried out in Italy at regional level allowed us to identify four different patterns, characterising different areas of the Country, which reflect different socio-economic conditions, labour market and welfare policies (see Fig 2).

Interestingly, the patterns identified by our analysis overlap under many aspects with those described by Bertin and Carradore [20]. The authors, analysing different welfare regimes in Italy, found substantial differences between the regions, describing heterogeneous welfare models which may account for disparities in social and health services. As a matter of fact, the data discussed regarding the extent of differences observed in regional social policies and welfare regimes in Italy have prompted the authors to reject the concept itself of a national welfare system and to describe a complexity which goes beyond the classical North-South social gradient [20]. The Italian welfare system picture, as reported by the authors, appears to be highly fragmented and its fragmentation is identified as one of the possible cause of social inequalities and disparity in health services available locally. The clusters identified help reconstructing a spectrum of regional realms ranging from “minimal” and more problematic systems (Campania, Puglia, Calabria), where social services are sustained by a limited number of both public and private providers, with lower level of assistance and insufficient policies innovation, to progressively more integrated systems, built on strong collaboration between public and Third sectors, coupled with innovative strategies and social policies (i.e. Emilia-Romagna, Toscana, Lombardia). At the opposite end of the spectrum, the welfare regimes can count on high presence of public and corporate actors, able to provide a widespread assistance and high social expenditure, but with fewer innovative social policies directed at safeguarding social inclusion (i.e. Bolzano-Trento, Valle d’Aosta) [20].

Similarly to what has been described [20], our findings indicate that in regions such as Emilia-Romagna, which historically positioned on the wealthier side of the Italian spectrum and benefits from an efficient welfare system, the overall trend observed in terms of unemployment rates, between people suffering from MHP and the rest of the population was very similar to that described at national level, albeit less pronounced (e.g. unemployment rates were lower than national data both in 2005 and 2013 in people with and without MHP).

A different scenario emerged from the analysis carried out in Regions such as Campania, where the comparison of unemployment rates between the groups, showed a parallel and worsening course during the crisis. In this case, we could assume that the proportionally equal increase of unemployment in both groups may be related to a historical structural, widespread deficiency of the labour market, which did determine an equal distribution of the problem between the two groups.

Another interesting and peculiar case is the one observed in the Region Basilicata, where almost paradoxically the unemployment rates increased significantly only for people non-suffering from MHP. This finding suggests a sort of unemployment “ceiling effect” for people who reported MHP which, in regions where the unemployment rate is already high, may eventually reach a “plateau” that remains stable despite the additional negative impact of the economic crisis.

An opposite scenario was observed in the province of Bolzano, one of the wealthiest area of Italy, where a significant divide between the two groups was found only with the beginning of the crisis (i.e. between 2005 and 2013). Although a possible explanation could be related to the “benefit trap” of welfare policies, which may reduce motivation to competitive labour market in people reporting MHP, resulting in the higher rates observed; these findings could also be ascribed to a possibly unbalanced labour market system, which, under economic pressure, tends to protect more efficiently the healthier group of the community. However, these data call for further investigation.

The study presents some limitations which require to be pointed out. First, data from the ISTAT surveys ‘Health Conditions and Use of Health Services’, carried out in 2004–2005 and 2012–2013, were not specifically collected for the purpose of this study and were not longitudinal in nature, as the same individuals were not interviewed in the two surveys. It is not possible to come to a definitive conclusion regarding the causal link between the variables explored. In particular, we cannot exclude that those subjects reporting MHP in the second survey were affected by MHP even prior to the crisis, thus, we cannot establish whether the reported MHP is a direct consequence of the crisis or not. The analysis carried out was limited to two time points and, although the impact of economic recession in 2013 appears quite robust, long-term effects cannot be investigated. In addition, the presence of a reverse causality (i.e. people unemployed are more likely to develop MHP) could not be verified.

A more general limitation, possibly causing underestimation of unemployment rates, is given by the definition adopted by ISTAT dataset, not including “inactive” subjects who might have been discouraged by the lower chance of finding a job and might have then ceased to be considered as part of the labour force.

Finally, the mental health index was derived from a self-reported instrument and not assessed by a clinician. However, the Mental Component Summary of SF-12 has been widely used and validated in population survey [25, 32].

In spite of these limitations, we believe that the present analysis may be of particular importance as it investigates the relationship between mental health and unemployment in Italy, a Country that shows important regional differences and has been strongly hit by the economic crisis.

The study also provides information on subgroups of individuals who reported MHP who may be particularly vulnerable during recession times. This may help planning wider and more accessible interventions aimed at supporting the most fragile groups in the community.

In conclusion, we believe that more attention should be given to reconsidering Mental Health policies towards these subgroups of vulnerable subjects. Amongst marginalised, unemployed people suffering from MHP, a negative and hopeless feeling may build up as a consequence of the crisis and this may prevent positive help-seeking behaviour.

Further research in this field is warranted to better evaluate the long-term effects of the economic recession on people who reported MHP.

## Supporting information

S1 Appendix Self-reported unemployment status among individuals with and without mental health problems (aged 18–64 years old) by regions in ISTAT 2005 and 2013. (DOCX)

S2 Appendix Backward stepwise selection. (PDF)
