# Supplementary material for: Self-reported unemployment status and recession: An analysis on the Italian population with and without mental health problems
Source: PLoS One. 2017 Apr 4;12(4):e0174135. doi: 10.1371/journal.pone.0174135 (PMC5380304; doi:10.1371/journal.pone.0174135)
Supplement: S1 Appendix — (DOCX) [file pone.0174135.s001.docx]

**Appendix**

Self-reported unemployment status among individuals with and without mental health problems (aged 18 – 64 years old) by Regions in ISTAT 2005 and 2013.

Source: our elaboration on the Surveys on Health and Use of Health Services, ISTAT 2005 and 2013
