# Supplementary material for: Self-reported unemployment status and recession: An analysis on the Italian population with and without mental health problems
Source: PLoS One. 2017 Apr 4;12(4):e0174135. doi: 10.1371/journal.pone.0174135 (PMC5380304; doi:10.1371/journal.pone.0174135)
Supplement: S2 Appendix — (PDF) [file pone.0174135.s002.pdf]

BACKWARD STEPWISE SELECTION

2004-2005

NO MENTAL HEALTH PROBLEMS

begin with full model

p < 0.1000 for all terms in model

Logistic regression	Number of obs	=	70461
	Wald chi2(6)	=	3075.94
	Prob > chi2	=	0.0000
Log pseudolikelihood = -7.058e+14	Pseudo R2	=	0.1356

disocc	Odds Ratio	Robust Std. Err.	z	P>|z|	[95% Conf.	Interval]
female	1.113308	.039351	3.04	0.002	1.038792	1.193169
age_gr	.5981874	.0125366	-24.52	0.000	.574114	.6232703
withkids	1.50773	.0778021	7.96	0.000	1.362699	1.668198
married	.4107111	.018724	-19.52	0.000	.3756045	.449099
istruzione	1.195974	.0256503	8.34	0.000	1.146742	1.247319
area	1.674298	.0329262	26.21	0.000	1.610991	1.740092
_cons	.0324144	.0032319	-34.39	0.000	.0266606	.03941

2004-2005

MENTAL HEALTH PROBLEMS

begin with full model

p = 0.9067 >= 0.1000 removing withkids

Logistic regression	Number of obs	=	10200
	Wald chi2(5)	=	364.65
	Prob > chi2	=	0.0000
Log pseudolikelihood = -1.249e+14	Pseudo R2	=	0.0772

disocc	Odds Ratio	Robust Std. Err.	z	P>|z|	[95% Conf.	Interval]
female	.5948426	.0486436	-6.35	0.000	.506751	.6982476
age_gr	.6862036	.0291451	-8.87	0.000	.6313933	.745772
area	1.474219	.060899	9.40	0.000	1.359564	1.598544
married	.5502048	.0533042	-6.17	0.000	.4550503	.6652568
istruzione	1.131377	.0485486	2.88	0.004	1.040115	1.230646
_cons	.1070511	.0166901	-14.33	0.000	.0788646	.1453115

2012-2013

NO MENTAL HEALTH PROBLEMS

begin with full model

p < 0.1000 for all terms in model

Logistic regression

Number of obs	=	61748
Wald chi2(6)	=	2738.15
Prob > chi2	=	0.0000
Pseudo R2	=	0.0838

Log pseudolikelihood = -1.119e+15

disocc	Odds Ratio	Robust Std. Err.	z	P>|z|	[95% Conf.	Interval]
female	1.09583	.0306106	3.28	0.001	1.037447	1.157499
age_gr	.67424	.0104138	-25.52	0.000	.6541351	.6949628
withkids	1.343467	.0485811	8.16	0.000	1.251546	1.44214
married	.5236628	.0185237	-18.29	0.000	.4885871	.5612566
istruzione	1.33395	.023389	16.43	0.000	1.288887	1.380588
area	1.349682	.0180844	22.38	0.000	1.314698	1.385596
_cons	.0867269	.0060437	-35.09	0.000	.0756548	.0994193

2012-2013

MENTAL HEALTH PROBLEMS

begin with full model

p = 0.1334 >= 0.1000 removing withkids

Logistic regression	Number of obs	=	10728
	Wald chi2(5)	=	370.99
	Prob > chi2	=	0.0000
Log pseudolikelihood = -2.506e+14	Pseudo R2	=	0.0482

disocc	Odds Ratio	Robust Std. Err.	z	P>|z|	[95% Conf.	Interval]
female	.6906631	.0400466	-6.38	0.000	.616469	.7737867
age_gr	.7282667	.0215124	-10.73	0.000	.6873006	.7716746
area	1.237503	.032764	8.05	0.000	1.174925	1.303415
married	.6793148	.0447737	-5.87	0.000	.5969917	.77299
istruzione	1.307344	.043711	8.02	0.000	1.224419	1.395886
_cons	.2270311	.0261821	-12.86	0.000	.1811012	.2846094
